# Tannin-Furanic Foams Formed by Mechanical Agitation: Influence of Surfactant and Ingredient Ratios

**DOI:** 10.3390/polym13183058

**Published:** 2021-09-10

**Authors:** Thomas Sepperer, Primož Šket, Alexander Petutschnigg, Nicola Hüsing

**Affiliations:** 1 Forest Products Technology and Timber Construction Department, Salzburg University of Applied Sciences, Markt 136a, 5431 Kuchl, Austria; alexander.petutschnigg@fh-salzburg.ac.at; 2 Salzburg Center for Smart Materials, Jakob-Haringer Straße 2a, 5020 Salzburg, Austria; 3 Slovenian NMR Center, National Institute of Chemistry, 1000 Ljubljana, Slovenia; primoz.sket@ki.se; 4 Department Chemistry and Physics of Materials, University of Salzburg, Jakob-Haringer Straße 2A, 5020 Salzburg, Austria; Nicola.Huesing@plus.ac.at

**Keywords:** natural polymer foam, pollutant absorption, blowing agent free, NMR, biopolymer, wastewater, poly(furfuryl alcohol)

## Abstract

With increasing demand of alternatives to oil-based lightweight materials, the development of tannin-based foams is getting more and more attention. In this paper, an alternative to traditionally used solvent-evaporation in the production of tannin-foams is presented. Mixing the tannin-furanic resin with different amounts of ionic and non-ionic surfactants at high agitational speed allows for the formation of highly porous, mechanically stable tannin-foams. Investigations on the influence of surfactant type and ingredient ratios on the foaming behavior and properties of the final foams were conducted. Materials obtained via this route do present extraordinary compression resistance (about 0.8 MPa), good thermal insulation (40 mW/m·K) and are suitable as a wastewater treatment agent at the end-of-life. It was shown that during mechanical blowing, homogeneous cross-sections and almost perfectly round pores form, leading to the high compression resistance. Investigations by means of Fourier transform infrared and ^13^C nuclear magnetic resonance spectroscopy show that the milder reaction environment leads to more linear poly(furfuryl alcohol)-tannin chains. This new type of tannin foam allows for use in various different fields of application ranging from durable building insulation to wastewater treatment.

## 1. Introduction

In the past years different research groups around the world presented multiple formulations towards tannin-based rigid foams. This interesting and almost completely bio-based [1] material has sparked the interest of the industry as an alternative to oil-based insulation foams [2,3], as well as due to their excellent thermal insulation values [4] and outstanding fire retardant behavior [5,6]. The majority of these foams is obtained by evaporation of a low boiling point solvent and simultaneous hardening of the polymer [7], which is in most cases is tannin-furfuryl alcohol, with [6,8,9,10,11] or without formaldehyde [2,5,12,13,14,15] or other hardeners [16,17]. Some studies were conducted using an oil emulsion templating method to produce monoliths [18] or to employ ethoxylated castor oil as the foaming agent [19,20]. One major drawback of these lightweight foams is their brittleness and relatively low mechanical stability. Although more stable foams can be produced by altering the acidic catalyst, blowing agent, or including additives [14], values higher than 0.2 MPa for compression resistance are hardly reported.

The two main components of tannin-furanic foams are, both bio-sourced, condensed tannin, and furfuryl alcohol. The first one, derived from various different species of trees, resembles the chemical structure and reactivity of resorcinol (in the condensed tannin A-ring) and pyrogallol (in the condensed tannin B-ring). The most common source for industrial extraction of condensed tannins are the acacia mearnsii (mimosa tannin) and the schinopsis balansae (quebracho tannin) tree. Tannin can easily be obtained from the bark and wood of these trees by hot-water extraction [21]. The second component, furfuryl alcohol, can be obtained by the acidic dehydration of hemi cellulose rich material (corn combs) into furfural and catalytic reduction into furfuryl alcohol [22].

In this work, the use of ionic and non-ionic surfactants as foaming agents in the mechanical agitation of mimosa tannin-furanic resin to form a porous, lightweight foam is reported. The influence of the type of surfactant type, its concentration, and the overall ration of the different ingredients on the foaming behavior and the chemical and physical properties of the tannin-furanic foams is thoroughly investigated. Alterations in the chemical composition of the tannin-furanic polymer are confirmed by FT-IR and ^13^C-NMR spectroscopy.

## 2. Materials and Methods

### 2.1. Chemicals and Reagents

Industrial mimosa tannin extract Weibul AQ was purchased from Tanac S.A. (Montenegro, Brazil). furfuryl alcohol (98%, technical) was provided by Transfurans Chemicals (Geel, Belgium). All other chemicals were purchased from VWR or Roth (Darmstadt, Germany). Sulfuric acid was diluted to a 2 mol/L solution in water, all other chemicals were used as received.

### 2.2. Foam Preperation and Design

Mechanically blown tannin-furanic foams were prepared by rapid agitation using an IKA (IKA, Staufen, Germany) overhead stirrer and a blade mixer. The general procedure included the following steps: (i) mixing of water, furfuryl alcohol, and surfactants, (ii) rapidly adding mimosa tannin and homogenizing by hand for 30 s, (iii) mounting to the overhead stirrer and homogenization at 200 rpm for 5 min, (iv) addition of acid and rapid agitation at 1500 rpm for 20 min. Ratios between tannin and furfuryl alcohol (T/F, 1–3.2), the amount of surfactant in relation to tannin and furfuryl alcohol and the ratio of tannin and acid (T/A 2.5–5) were varied to evaluate suitable values. Water was added at a level of 60% of the tannin to allow for a lower viscosity and good dissolution. Five different surfactants were used: (i) Sodiumdodecyl sulfate (anionic), (ii) TritonX-100 (non-ionic), (iii) Tween-80 (non-ionic), (iv) Brij 35 (non-ionic), and (v) Pluronic F127 (non-ionic).

After the foam formed a light-brown, airy looking wet-foam-mass, it was transferred into PTFE-coated moulds and cured in a convection oven at 70–90 °C for half an hour before it was removed from the mould and the skin was peeled off. Final drying was performed for another 24 h at 70 °C.

### 2.3. Physical Characterization

Bulk density was calculated by dividing the sample mass through its volume, and skeletal density was evaluated by using a helium pycnometer UltraPyc 1200 e (Quantachrome, Graz, Austria). To do so, the foams were ground into fine powder and placed in the measuring cell. The cell was purged for 1 min under constant flow of helium. Target pressure was set to 12 psi and the average of 10 measurements was calculated. Cell size and orthotropy were collected and calculated using a Nikon SMZ 1500 transmitted light microscope (Nikon, Tokio, Japan), by conducting at least 50 measurements for each foam as described previously [14]. Scanning electron microscopy (SEM) images were taken using a Zeiss Ultra Plus field emission scanning electron microscope equipped with an annular backscatter electron detector (Carl Zeiss AG, Oberkochen, Germany). The acceleration voltage was set to 5 kV and the working distance was adjusted between 4 and 6 mm. Prior to the imaging, the samples were coated with a thin layer of gold using a sputter coater with a current of 40 mA and coating time of 60 s. Thermal Conductivity of the foams was measured using a λ-meter EP500e (Lambda-Messtechnik, Dresden, Germany) at 10, 25, and 40 °C. The temperature difference between upper and lower plate was 10 °C.

### 2.4. Chemical Characteristics

Attenuated total reflectance Fourier-transform infrared (ATR FT-IR) spectra were collected using a Perkin-Elmer (Perkin-Elmer, Waltham, MA, USA) Frontier FT-IR spectrometer, equipped with an ATR Miracle accessory. Thirty-two scans were performed for each sample, at a resolution of 4 cm^−1^, in the range 4000–600 cm^−1^, and Bio Rad KnowItAll (BioRad, California, CA, USA) software was used for normalizing and averaging.

^13^C-CP-MAS NMR spectra of solid samples were recorded on a Bruker Avance NEO 400 MHz NMR spectrometer equipped with 4 mm CP-MAS probe. Larmor frequencies of carbon and proton nuclei were 100.626 MHz and 400.142 MHz. The ^13^C CP-MAS NMR spectra were externally referenced using adamantane. All samples were spun at the magic angle with 15 kHz during ^13^C measurements. The pulse sequence used for acquiring the ^13^C spectra was a standard cross-polarization MAS pulse sequence with high-power proton decoupling was used during acquisition. The repetition delay in all experiments was 5 s.

To estimate the amount of acid used up in the polymer formation, the acid recovery was calculated as described previously by the group [14]. Briefly, 5 g of the dried and ground foam were washed three times with 250 mL water. The washings were combined and made up to 1 L with water. The pH of the solution was recorded with an inoLab 720 pH 720 m (Xylem, New York, NY, USA). Weight of the foams after rinsing and drying was recorded, and leaching has been calculated.

Pollutant absorption experiments on the mechanically blown tannin foams were done as described previously by the group [23]. Substances used to simulate pollutants were cationic dye methylene blue and non-ionic molecule riboflavin. Briefly, in a solution containing 10 ppm pollutant, washed (with water and ethanol, 3 times each) and dried tannin foam was added in an equivalent of 1 mg foam per 5 mL solution. Afterwards, magnetic stirring was applied for 48 h in the dark. Concentration of riboflavin and methylene blue were determined using a VWR P4 spectrophotometer (VWR, Ismaning, Germany). To observe the kinetics of the absorption during the first 6 h, spectra in the range of 300–800 nm were recorded in 30 min intervals. During the kinetics study a concentration of 1 mg foam per mL of solution was used. Before measuring, the solutions were centrifuged at 4000 rpm for 3 min and the concentration of pollutants was calculated with the absorbance at 444 nm for riboflavin and 664 nm for methylene blue. Absorption capacity q was calculated according to Equation (1), where *q* is the absorption capacity, *C_0_* an *C_t_* are the initial and equilibrium concentration (ppm) of the pollutant, *V* is the volume of the pollutant solution (mL), and *W* the amount of absorbent used (mg). Results are presented as mg pollutant absorbed per *g* of absorbent.

(1) $$\frac{\left(C_{0}-C_{t}\right)\times V}{W}.$$

## 3. Results

In the following paragraphs, the results are presented. Percent values are always expressed as wt/wt. Surfactant concentration is expressed as wt. −% based on the weight of tannin and furfuryl alcohol (which make up the majority of the hardened polymer). Foams are labeled as surfactant type and its concentration in wt.−% based in the final polymer. (e.g., a foam made from TWEEN 80 with a concentration of 6% surfactant based on the cured polymer is labeled TWEEN 6)

### 3.1. Variations of the Surfactant as Well as Acid and Furfuryl Alchohol Ratios

**Figure 1:**
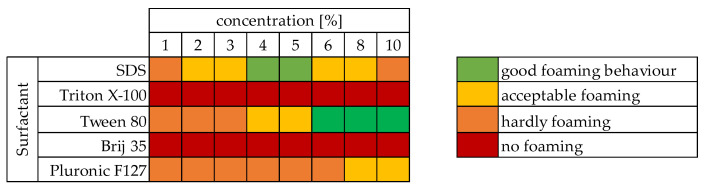
Heatmap of foaming behavior in dependence of surfactant type and concentration.

Variations of the type of surfactant as well as its concentration were performed using 40 g of tannin, a T/F-ratio of 1.6, and a T/A-ratio of 2.5. Results of these investigations are shown in form of a heat map in Figure 1, indicating the quality from the foams obtained by mechanical agitation. Depending on the volume increase after 20 min of agitation, the mixtures were rated. When no, or almost no volume increase (less than 1.5× the starting volume) was observed, it was reported as no or hardly foaming. If a volume increase of more than 4× was observed, a good foaming behavior was reported. After stopping the agitation, the foam mass should be stable for at least 15 min to account for acceptable foaming, as this is the minimum time required to get initial hardening in the oven. If the mass was stable for longer, good foaming was reported.

Best results in terms of foam stability, homogeneity, and drying behavior were observed for 10% TWEEN-80 and 4% SDS. Other concentrations of these two surfactants did produce a foam-like wet mass, which was either too dense (SDS below 4% and TWEEN below 4%) or too airy; the latter samples collapsing a few seconds after stopping the agitation (SDS 10%). Whilst Pluronic F127 did lead to some foaming in all concentrations, the material obtained was very dense and therefore not suitable for production of tannin-furanic foams. For both non-ionic surfactants Triton X-100 and Brij 35, no foaming was observed. It was expected that Pluronic will lead to less airy foams as it is mostly used in emulsions to prevent foaming yet forming homogeneous sub structures [24]. As TWEEN does not present the best foaming capacities, it does, however, present great properties as an emulsifier and therefore ensuring homogeneous distribution of the main components within the final product. Foams obtained from SDS (2–8%) were stable after stopping the agitation for about 15 min giving enough time for the polymer to cure before it collapses. The TWEEN (4–10%) containing formulations were stable at room temperature for over an hour, again giving it enough time to harden in the oven before the structure loses volume. Depending on the structure (longest carbon chain) of the surfactant, linearity and charge, different foaming abilities, or volume increases were observed for the tannin-furanic mixtures. This behavior was already reported in the past, where anionic surfactants lead to the greatest volume increase, while non-ionic surfactants showed better foam stability, yet less volume increase [25].

**Figure 2:**
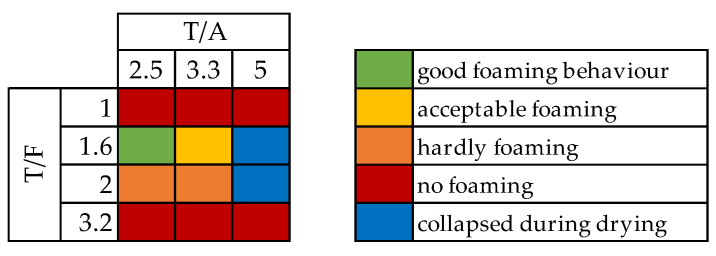
Heatmap of foaming behavior depending on T/F and T/A ratio using 4% SDS.

Another tool in the optimization of the foam composition is to alter the ratio of tannin to furfuryl alcohol (T/F) and tannin to acid. For these optimizations SDS 3–6% and TWEEN 6–10% foams were produced. Results for using 4% SDS are presented as a heat map in Figure 2.

For all surfactants and concentrations, results were quite similar. The only difference was that for SDS at a T/A ratio of 5 the foams did not harden at all, and a liquid was left in the oven. Meanwhile, for TWEEN at a T/A ratio of 5, a foam was obtained, yet only after letting it sit in the oven for over an hour within the mould. These foams were also much more compact and denser than the others.

Further foams in this study were all made with a T/F ratio of 1.6 and a T/A ratio of 2.5. Room temperature during foam preparation was constantly between 20 and 22 °C. With further increase, the environment temperature and the amount of acid or water might have to be adjusted.

### 3.2. Physical Characterisation and Properties of the Resulting Foams

**Figure 3:**
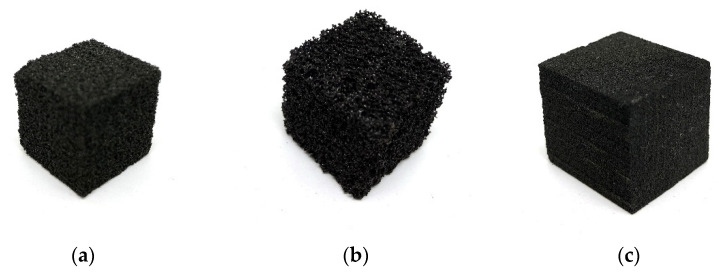
Photographs of (**a**) SDS 4 foam; (**b**) SDS 6 foam and (**c**) TWEEN 10 foam.

As determined beforehand, only the surfactants SDS and TWEEN resulted in acceptable foams. For all further tests, only foams made with 3 to 6% SDS and 6 to 10% TWEEN were considered. Generally, all foams produced by the mechanical beating method do present an isotropic and homogeneous macro-pore shape, as well as a quite rigid and not very brittle appearance. In Figure 3, photographs of SDS 4, SDS 6, and TWEEN 10 are presented. Overall, TWEEN 80 did lead to the formation of smaller pores, yet with increasing concentration, the pore size decreased while for SDS a positive dependence between surfactant concentration and pore size was observed.

**Figure 4:**
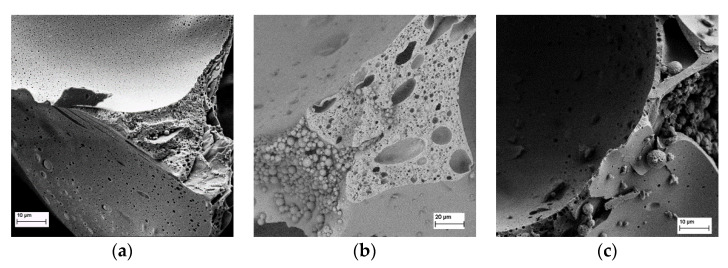
Photographs of (**a**) tannin foam obtained by solvent evaporation; (**b**) SDS 4 foam and (**c**) TWEEN 10 foam.

A closer look at the different microstructures caused by the surfactants is illustrated in Figure 4. The SEM micrographs show small sections of a tannin foam produced by solvent evaporation, a SDS-based one and a TWEEN-based one, the first one having numerous very small pores scattered across the cell walls, while the SDS-based one presents bigger pores within the cell walls and thicker walls in general. For TWEEN, hardly any cavities within the cell walls are present. Using surfactants for mechanically beaten foams also leads to the formation of small beads that stick to the cell walls and cavities within. For both surfactants an increase in wall thickness compared to solvent-blown foams was observed.

Results for physical properties are listed in Table 1, where ρ_bulk_ is the bulk density of the foams, θ presents the porosity, and σ_c_ is the compression resistance. Other values are pore diameter and orthotropy. All foams produced with surfactants and mechanical agitation presented a homogeneous cross-section and almost perfectly round pores (orthotropy for all below 1.08). By changing the curing temperature for SDS-based foams from 90 °C to 70 °C, evaporation from water during the drying could be mitigated and therefore had a positive influence on the spherical shape of the pores. The most interesting properties for tannin-furanic foams are their density and compression resistance. Although the density of the TWEEN-based foams is about 4–5 times higher compared to foams obtained by the solvent-evaporation, their compression resistance increased by at least one order of magnitude. The same is true for SDS-based foams, where the density was roughly 2–3 times higher, compared to the classical tannin-furanic foam materials. The overall increase in compression resistance despite the higher density is clearly shown by the relative compression resistance σ_rel_ (σ_c_/ρ_bulk_). σ_rel_ is very much constant for the SDS-based foams at around 3.8, and therefore already higher compared to foams made by solvent evaporation (0.8–2.8). For the TWEEN-based foams, with increasing TWEEN concentration, σ_rel_ increased from 2.4 to 4.1, once more due to the inverse foaming ability of TWEEN. Compared to commercially available polyurethane foams (35 kg/m^3^, σ_c_ 0.15 N/mm^2^), compression resistance of mechanically blown tannin-furanic foams is comparable or even higher (https://daemmt-besser.de/pu-daemmstoffe/eigenschaften (accessed on 6 September 2021)). Yet compression resistance does not reach the polymethacrylimide foam (density 32–170 kg/m^3^ and σ_c_ 0.4–3.2 N/mm^2^) (https://www.swiss-composite.ch/pdf/t-Rohacell-d-f.pdf (accessed on 6 September 2021)).

Besides due to the density increase, the overall enhancement in mechanical stability of the tannin-furanic foams can be traced back to the thicker cell walls as shown in the SEM (Figure 4b,c). Compared to classical tannin foams (Figure 4a) the cell walls of mechanically formed foams are thicker, less perforated, and therefore more stable.

To evaluate the influence of surfactant concentration on foam properties, linear regression analysis was performed, and the results are presented in Table 2. As the concentration of surfactant does have a significant influence on the density of the foams, all other properties influenced by the density are also influenced the amount of surfactant used. With increasing SDS concentration, density and therefore also compression resistance decreased, while for TWEEN the opposite was observed. These results are expected, as higher amounts of TWEEN do inhibit the foaming [26].

**Figure 5:**
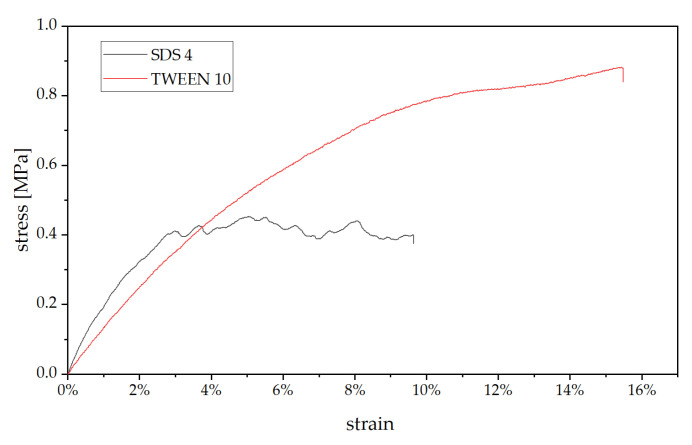
Stress-strain graph for SDS 4 and TWEEN 10.

All SDS foams did show a stress-strain curve typical for porous material, where after the linear elastic region, multiple breaks can be observed, corresponding to failure of cell walls, while the TWEEN-based foam showed a compression behavior more associated with non-porous solids (Figure 5). The higher compression resistance could be explained by (i) higher density and (ii) thicker cell walls combined with less cavities as seen in the SEM, leading to a more stable microstructure.

Porosity was lower for the TWEEN-based foams, which could already be deducted from the SEM micrographs in Figure 4. Once more the foaming inhibition character of TWEEN comes into place as higher concentrations lead to reduced porosity. SDS-based foams showed a porosity of roughly 90%, which is lower compared to foams obtained by solvent evaporation, as less cavities form during the mechanical foaming procedure.

Thermal conductivity was measured at samples with dimensions of 250 × 250 × 50 mm^3^. For SDS-based foams the formulation using 4% surfactant was tested and showed a thermal conductivity of roughly 39 mW/m·K and therefore is in the range of foams made by solvent-evaporation [14] and tannin-hexamine mechanically foamed ones [20]. The thermal conductivity of foams produced with 10% TWEEN was roughly 45 mW/m·K, which is a relatively good result for the high density and could be partially explained by the small pore diameter (~80 µm).

### 3.3. Chemical Characteristics and Properties

**Figure 6:**
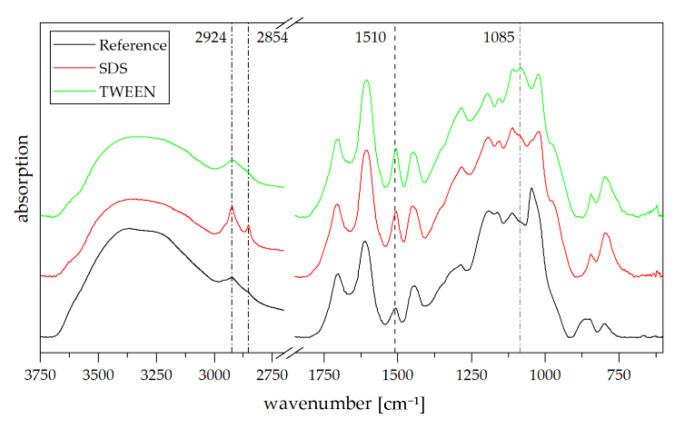
FT-IR spectra of rinsed SDS and TWEEN-based tannin foams. Classic tannin foam is given as reference.

Peak assignment for the ATR FT-IR spectra of formaldehyde free tannin-furanic foams were discussed extensively in the literature [27]. Compared to foams obtained by solvent evaporation, additional peaks in the antisymmetric (2924 cm^−1^) and symmetric (2854 cm^−1^) C-H stretching region can be observed in the SDS foam (Figure 6). As these peaks are still present after rinsing with water and ethanol, it is likely that SDS shows chemical interactions with tannin in a similar way as it does with phenol [28,29]. Increased peak intensity of the C=C stretching vibration at 1510 cm^−1^ might indicate an increased presence of 2,5-disubstituted furan ring [30]. The additional signal at 1085 cm^−1^ corresponds to residues of TWEEN 80.

**Figure 7:**
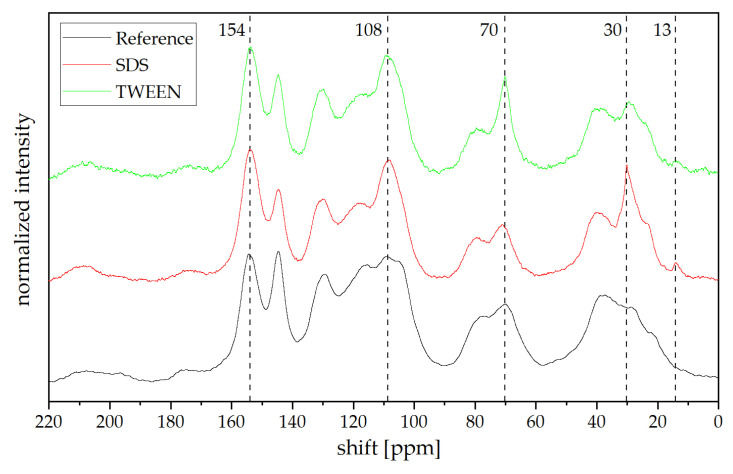
^13^C CP MAS NMR spectra of reference foam, SDS 4, and TWEEN 10 foams.

^13^C CP MAS NMR spectra for a solvent-blown tannin foam (the reference foam), as well as a TWEEN-based and SDS-based foam are presented in Figure 7. Although thorough rinsing of the foams with water and ethanol was performed prior to the analysis, signals corresponding to SDS and TWEEN are still present. The signal at 13 ppm corresponds to CH_3_ and the one at 30 ppm to the aliphatic CH_2_ moieties of the SDS or TWEEN; the more intense signal at 70 ppm can be assigned to CH groups in position 2 of the flavonoid C-ring [8] and the R-CH_2_-O moieties in TWEEN. Compared to the standard tannin-furanic foam, the mechanically foamed ones present a higher intensity of signals at 108 and 154 ppm, both corresponding to C3/C4 and C2/C5 in the furan ring, respectively [30]. Combined with a higher intensity of the peak at 1510 cm^−1^ from the FT-IR analysis, it suggests less ring-opening taking place during formation of the tannin-furfuryl polymer. The reaction conditions do have great influence on the structure and chemical formation of poly(furfuryl alcohol), as shown in the past [31]. Overall reaction conditions during mechanical foaming are much less severe (in terms of acidity and time) compared to the classical solvent-evaporation approach, that more linear chains of poly(furfuryl alcohol) can be formed between the flavonoid units of tannin. Furthermore, through the increased time of homogenization (complete time when foaming at ambient temperature) a more homogeneous distribution can be achieved compared to the classical approach.

**Figure 8:**
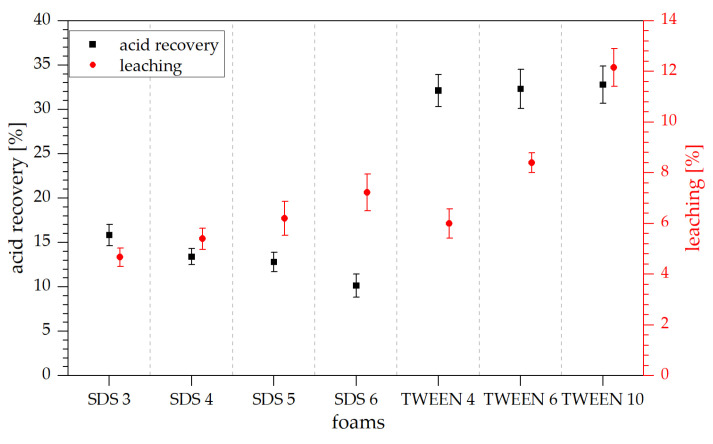
Acid recovery and leaching results of the tannin foams.

For classical formaldehyde-free tannin-furanic foams catalyzed by sulfuric acid, around 30 to 40% of the acid remain in the final foam and can be recovered by washing. In case of the TWEEN-based foams, roughly 33% of the catalyst were recovered (Figure 8), which indicates a similar behavior as reported in literature. During leaching, the TWEEN-based foam loses between 6 and 12% of its dry weight. This agrees well with the surfactant released, the acid recovered, and some unreacted furfuryl alcohol. Assuming that only little of the surfactant remains in the polymer after leaching, around 1% of the furfuryl alcohol is leached from the final foam.

For the SDS-based foams, leaching does also increase with increasing surfactant concentration, while acid recovery decreases from 16 to 10%. The decrease in acid recovery with the increasing amount of surfactant might be due to the partial neutralization of the oxonium ions from the sulfuric acid by the anionic head group in SDS [32,33]. Hence, the more anionic surfactant is present in the foams, the higher more acid can be neutralized.

Table 3 shows a summary of the absorption capacity for methylene blue and riboflavin for the different foams as well as the separation efficiency. It was shown earlier by members of the group that tannin foams made by solvent evaporation absorb up to 63 mg methylene blue per *g* of absorbent, on average around 45 mg/g. Foams investigated in this paper did absorb less of the cationic dye, around 34 mg/g for the TWEEN-based foams and around 27 mg/g for the SDS-based ones. No difference could be observed for surfactant-concentration variation, but for the type of surfactant. The lower absorption capacity for the SDS foams might come from a partial saturation with charged SDS on the functional groups active in methylene blue absorption. This would also explain the aliphatic C-H signal in the FT-IR spectra. The overall lower decrease in capacity could be due to the lower porosity of the foams concomitant with a smaller available active surface area.

When performing the absorption experiments in a mixture of methylene blue and riboflavin, the material tends to favor the absorption of the cationic dye even more. In separate runs, the riboflavin absorption capacity for TWEEN 10 is around 4.08 mg/g, while in runs where the two pollutants are mixed, riboflavin absorption capacity decreases to 2.38 mg/g. This effect is also true for the SDS foams, yet the changes are not that big.

**Figure 9:**
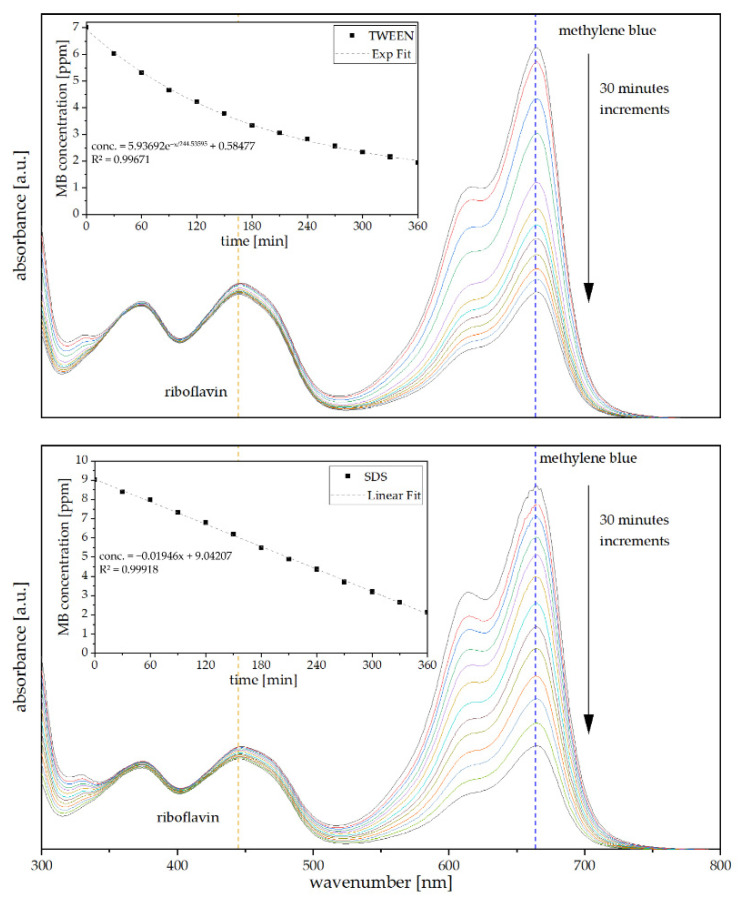
UV/vis spectra of a kinetic study on a MB/Riboflavin mixture over 360 min. TWEEN 10 (**top**) and SDS 4 (**bottom**) as well as fitted curves for absorption model prediction.

Figure 9 shows the UV/vis spectra in a range of 300–800 nm over the course of 6 h every 30 min while treating either with TWEEN 10 foam (top panel) or SDS 4 foam (bottom panel) as well as the corresponding methylene blue concentration. Initial MB concentration was 9 ppm for both foams, yet measuring MB concentration in the mixture containing TWEEN-based foam right after addition a sharp reduction to around 7 ppm was observed. While for the TWEEN-containing foam, absorption is rapid in the beginning, but it starts to slow down over time. For SDS-based foams, the absorption is constant at 0.0019 mg methylene blue per minute. Nevertheless, it is clearly visible for both foams that the signal at 664 nm, corresponding to methylene blue decreases while the riboflavin signal at 444 nm remains almost constant. These results suggest that tannin-furanic foams are suitable for the separation of charged and non-charged pollutants in water, opening up for new possible applications.

## 4. Conclusions

Numerous variations of lightweight tannin-based foams have been investigated over the past years, all of them still facing major challenges concerning their brittleness and sometimes fragility. The tannin-furanic foams obtained by mechanical agitation in the presence of different surfactants and concentrations presented in this article show excellent mechanical properties even when considering their higher density compared to foams obtained by solvent evaporation. They do present a homogeneous cross-section of the cell walls with pores almost perfectly spherical shaped. Although their thermal conductivity is higher compared to classical tannin-based foams, they are comparable to other bio-based insulation material such as wood-fiber boards with a similar density. It was confirmed by FT-IR and ^13^C CP MAS NMR spectroscopy that the type of entanglements of the tannin-furanic polymer changes slightly in the mechanical foaming procedure compared to the classical solvent-evaporation approach. This could be due to changes in the reaction environment (less acidic, lower temperature, longer homogenization). Foams made with SDS as a surfactant present a less acidic character due to the partial neutralization reactions of the acid with the anionic head group of the surfactant.

Exploring alternative use-cases or end-of-life scenarios for these foams, showed excellent selectivity for cationic dye removal from a mixture of cationic and non-ionic pollutants. The homogeneity and high mechanical resistance of foams made with TWEEN have already aroused interest in the industry and can provide a suitable and resistant alternative to polyurethane or polystyrene foams. Furthermore, this family of foams would be suitable for insulation not only in walls but also in flooring or other regions, where pressure is applied.

## Figures and Tables

**Table 1:** Results for mechanically beaten foams. Mean values and standard deviation in brackets.

Scheme 3		ρ_bulk_ [g/cm^3^]	θ	σ_c_ [N/mm^2^]	Pore Ø [µm]	Ortho.
SDS	3	0.124 (0.0026)	0.897 (0.0022)	0.479 (0.024)	322.2 (6.985)	1.076 (0.019)
	4	0.111 (0.0028)	0.907 (0.0024)	0.429 (0.011)	335.7 (3.499)	1.072 (0.035)
	5	0.105 (0.0027)	0.914 (0.0023)	0.398 (0.009)	387.9 (4.068)	1.056 (0.186)
	6	0.102 (0.0036)	0.916 (0.0030)	0.355 (0.017)	424.7 (3.309)	1.064 (0.037)
TWEEN	4	0.143 (0.0033)	0.882 (0.0027)	0.337 (0.016)	112.2 (0.921)	1.032 (0.023)
	6	0.152 (0.0023)	0.875 (0.0019)	0.464 (0.023)	94.9 (1.211)	1.034 (0.015)
	10	0.171 (0.0013)	0.857 (0.0011)	0.843 (0.053)	81.2 (1.469)	1.016 (0.01)

**Table 2:** Coefficient of determination R^2^, significance level and Pearson’s *r* from linear regression analysis.

Surfactant		ρ_bulk_	θ	σ_c_	Pore Ø	Ortho.
SDS	R^2^	0.803 *	0.842 *	0.886 *	0.946 *	0.038
	significance level	0.000	0.000	0.000	0.000	0.408
	Pearson’s *r*	−0.896	0.918	−0.941	0.973	−0.196
TWEEN	R^2^	0.959 *	0.960 *	0.967 *	0.928 *	0.151
	Significance level	0.000	0.000	0.000	0.000	0.152
	Pearson’s *r*	0.979	−0.9799	0.983	−0.963	−0.389

A single asterisk indicates a significance level of alpha <0.001.

**Table 3:** Results of the absorption experiments, standard deviation in brackets.

Surfactant		MB Absorption Capacity [mg/g]	Riboflavin Absorption Capacity [mg/g]	Separation Efficiency [%]
SDS	3	27.57 (2.337)	3.97 (0.457)	88.97 (1.931)
	4	27.04 (1.995)	3.75 (1.067)	90.02 (2.692)
	5	27.44 (2.255)	3.29 (1.752)	89.16 (0.937)
	6	28.59 (2.002)	3.35 (1.551)	90.26 (1.091)
TWEEN	4	34.08 (0.815)	4.35 (0.992)	92.99 (0.379)
	6	33.79 (1.390)	4.10 (0.297)	93.69 (0.942)
	10	34.12 (1.430)	4.08 (0.280)	93.56 (0.269)

## Data Availability

Not applicable.

## References

[1] Basso M.C., Giovando S., Pizzi A., Celzard A., Fierro V. (2013). Tannin/furanic foams without blowing agents and formaldehyde. Ind. Crops Prod..

[2] Tondi G., Link M., Kolbitsch C., Lesacher R., Petutschnigg A. (2016). Pilot plant up-scaling of tannin foams. Ind. Crops Prod..

[3] Martinez de Yuso A., Lagel M.C., Pizzi A., Fierro V., Celzard A. (2014). Structure and properties of rigid foams derived from quebracho tannin. Mater. Des..

[4] Basso M.C., Li X., Fierro V., Pizzi A., Giovando S., Celzard A.G. (2011). Formaldehyde-free, Foams For Thermal Insulation. Adv. Mater. Lett..

[5] Kolbitsch C., Link M., Petutschnigg A., Wieland S., Tondi G. (2012). Microwave Produced Tannin-furanic Foams. J. Mater. Sci. Res..

[6] Tondi G., Zhao W., Pizzi A., Du G., Fierro V., Celzard A. (2009). Tannin-based rigid foams: A survey of chemical and physical properties. Bioresour. Technol..

[7] Pizzi A. (2019). Tannins: Prospectives and Actual Industrial Applications. Biomolecules.

[8] Tondi G., Pizzi A. (2009). Tannin-based rigid foams: Characterization and modification. Ind. Crops Prod..

[9] Li X., Pizzi A., Lacoste C., Fierro V., Celzard A. (2012). Physical Properties of Tannin/Furanic Resin Foamed With Different Blowing Agents. BioResources.

[10] Meikleham N.E., Pizzi A. (1994). Acid- and alkali-catalyzed tannin-based rigid foams. J. Appl. Polym. Sci..

[11] Marie Z., Nicolas V., Celzard A., Fierro V. (2019). Experimental investigation of the physical foaming of tannin-based thermoset foams. Ind. Crops Prod..

[12] Tondi G., Petutschnigg A. (2017). Tannin-based foams: The innovative material for insulation purposes. Handb. Compos. Renew. Mater..

[13] Link M., Kolbitsch C., Tondi G., Ebner M., Wieland S., Petutschnigg A. (2011). Formaldehyde-free tannin-based foams and their use as lightweight panels. BioResources.

[14] Eckardt J., Neubauer J., Sepperer T., Donato S., Zanetti M., Cefarin N., Vaccari L., Lippert M., Wind M., Schnabel T. (2020). Synthesis and Characterization of High-Performing Sulfur-Free Tannin Foams. Polymers.

[15] Lacoste C., Čop M., Kemppainen K., Giovando S., Pizzi A., Laborie M.-P.P., Sernek M., Celzard A. (2015). Biobased foams from condensed tannin extracts from Norway spruce (Picea abies) bark. Ind. Crops Prod..

[16] Lacoste C., Basso M.C., Pizzi A., Laborie M.P., Garcia D., Celzard A. (2013). Bioresourced pine tannin/furanic foams with glyoxal and glutaraldehyde. Ind. Crops Prod..

[17] Lacoste C., Basso M.C., Pizzi A., Celzard A., Laborie M.-P. (2015). Natural albumin/tannin cellular foams. Ind. Crops Prod..

[18] Szczurek A., Martinez de Yuso A., Fierro V., Pizzi A., Celzard A. (2015). Tannin-based monoliths from emulsion-templating. Mater. Des..

[19] Santiago-Medina F.J.J., Delgado-Sánchez C., Basso M.C.C., Pizzi A., Fierro V., Celzard A. (2018). Mechanically blown wall-projected tannin-based foams. Ind. Crops Prod..

[20] Celzard A., Stauber M., Szczurek A., Fierro V., Pizzi A. (2014). A new method for preparing tannin-based foams. Ind. Crops Prod..

[21] Pizzi A. (2008). Tannins: Major Sources, Properties and Applications. Monomers, Polymers and Composites from Renewable Resources.

[22] Wang H., Yao J. (2006). Use of Poly(furfuryl alcohol) in the Fabrication of Nanostructured Carbons and Nanocomposites. Ind. Eng. Chem. Res..

[23] Sepperer T., Neubauer J., Eckardt J., Schnabel T., Petutschnigg A., Tondi G. (2019). Pollutant Absorption as a Possible End-Of-Life Solution for Polyphenolic Polymers. Polymers.

[24] Braghiroli F.L., Fierro V., Parmentier J., Pasc A., Celzard A. (2016). Easy and eco-friendly synthesis of ordered mesoporous carbons by self-assembly of tannin with a block copolymer. Green Chem..

[25] Wang H., Guo W., Zheng C., Wang D., Zhan H. (2017). Effect of Temperature on Foaming Ability and Foam Stability of Typical Surfactants Used for Foaming Agent. J. Surfactants Deterg..

[26] Kothekar S.C., Ware A.M., Waghmare J.T., Momin S.A. (2007). Comparative Analysis of the Properties of Tween-20, Tween-60, Tween-80, Arlacel-60, and Arlacel-80. J. Dispers. Sci. Technol..

[27] Tondi G., Link M., Oo C.W., Petutschnigg A. (2015). A Simple Approach to Distinguish Classic and Formaldehyde-Free Tannin Based Rigid Foams by ATR FT-IR. J. Spectrosc..

[28] Zhang Y., Cao W., Xu J. (2002). Multilayer Films from Phenolic Resin–Sodium Dodecyl Sulfate Complex and Polycations. J. Colloid Interface Sci..

[29] Zhang Y., Cao W. (2000). A novel photosensitive ternary complex consisting of phenol-formaldehyde resin, sodium dodecyl sulfate, and diazo resin. J. Polym. Sci. Part A Polym. Chem..

[30] Tondi G., Cefarin N., Sepperer T., D’Amico F., Berger R.J.F., Musso M., Birarda G., Reyer A., Schnabel T., Vaccari L. (2019). Understanding the Polymerization of Polyfurfuryl Alcohol: Ring Opening and Diels-Alder Reactions. Polymers.

[31] Sadler J.M., Yeh I., Toulan F.R., McAninch I.M., Rinderspacher B.C., La Scala J.J. (2018). Kinetics studies and characterization of poly(furfuryl alcohol) for use as bio-based furan novolacs. J. Appl. Polym. Sci..

[32] Kartal Ç., Akbaş H. (2005). Study on the interaction of anionic dye–nonionic surfactants in a mixture of anionic and nonionic surfactants by absorption spectroscopy. Dye. Pigment..

[33] Nesterenko P.N., Haddad P.R., Hu W. (2003). Studies on the separation of hydronium ion using surfactant-modified reversed-phase stationary phases and eluents containing an acidified electrolyte. J. Chromatogr. A.

